# Microplastic Polymer Mass Fractions in Marine Bivalves: From Isolation to Hazard Risk

**DOI:** 10.3390/jox15060186

**Published:** 2025-11-06

**Authors:** Tanja Bogdanović, Irena Listeš, Jennifer Gjerde, Sandra Petričević, Zvonimir Jažo, Eddy Listeš, Jelka Pleadin, Darja Sokolić, Ivona Jadrešin, Federica di Giacinto

**Affiliations:** 1 Croatian Veterinary Institute, Regional Veterinary Institute Split, 21000 Split, Croatia; t.bogdanovic.vzs@veinst.hr (T.B.); petricevic.vzs@veinst.hr (S.P.); jazo.vzs@veinst.hr (Z.J.); e.listes.vzs@veinst.hr (E.L.); i.jadresin.vzs@veinst.hr (I.J.); 2 Institute of Marine Research, 5817 Bergen, Norway; jennifer.gjerde@hi.no; 3 Laboratory for Analytical Chemistry, Croatian Veterinary Institute, 10000 Zagreb, Croatia; pleadin@veinst.hr; 4 The Faculty of Tourism and Rural Development in Požega, 34000 Požega, Croatia; darja.sokolic@gmail.com; 5 Istituto Zooprofilattico Sperimentale dell’Abruzzo e del Molise “G. Caporale”, 64100 Teramo, Italy; f.digiacinto@izs.it

**Keywords:** marine bivalves, microplastics, pyrolysis gas chromatography–mass spectrometry (Pyr-GC/MS)

## Abstract

Microplastics (MPs) are a ubiquitous marine pollutant, and their presence in bivalves is receiving increasing attention due to the associated risks to human health. The steps of pretreatment, detection, and quantification in the analysis of MPs depend on the type of polymer. Research on MPs is challenging because of the varying characteristics of these materials, such as the size, shape, and polymer type. Consequently, there are no standardized methods for their collection, separation, identification, or quantification. This review specifically examines the available bivalve digestion steps, focusing on efficient and time-reducing methods, such as the microwave-assisted (MAW) procedure and its advantages. Recent achievements in the application of pyrolysis gas chromatography–mass spectrometry (Pyr-GC-MS) are presented for the profiling of polymer mass-related microplastics data in marine bivalves. Here, we provide an overview of the abundance, properties, and polymer types of MPs in bivalve species, highlighting the polymer mass fractions. To date, the available mass-based concentrations have revealed nine types of MPs—polyethylene (PE), polypropylene (PP), polyvinyl chloride (PVC), polyethylene terephthalate (PET), polystyrene (PS), polymethyl methacrylate (PMMA), polyamide 66 (PA66), polycarbonate (PC), and polyamide 6 (PA6)—with PE, PP, and PVC being the most common. The total MP levels in bivalves were at ppm levels, ranging from 0.26 µg/g to 36.4 µg/g wet weight. The risk of human ingestion of MPs was assessed through the consumption of bivalves as seafood. The overall potential human health risk value (H) for marine bivalves was classified within the moderate to high hazard category.

## 1. Introduction

Microplastics (MPs) are a subgroup of polymers characterized by a particular physical form. They can be primary MPs, i.e., substances intentionally introduced into products, or secondary MPs, i.e., substances that are created by abrasion from objects [1]. Plastics make up most of the litter in the marine environment [2] and are present in all terrestrial environments [3]. In general, plastics found in the environment are classified according to their size: macroplastics are larger than 25 mm; mesoplastics are between 5 mm and 25 mm; MPs measure between 5 mm and 1 µm; and nanoplastics are smaller than 1 µm [4,5,6,7,8]. MPs are multidimensional pollutants that differ in size, shape, polymer type, and chemical composition. Each of these dimensions can influence the toxicity of the particle.

The polymer type has also been identified as a potential factor influencing toxicity, as some polymer types are composed of monomers that are inherently more toxic than others, in addition to the specific additives associated with each polymer type as an end product. Lithner et al. classified polymer types according to the hazardousness of their monomers and found that polypropylene (PP) and polyethylene (PE) are among the least hazardous, while polyvinyl chloride (PVC) and polystyrene (PS) are among the most hazardous [9]. The most commonly used commercial polymers are polypropylene (PP), polyethylene (PE), high-density polyethylene (HDPE), low-density polyethylene (LDPE), linear low-density polyethylene (LLDPE), polyvinyl chloride (PVC), polyurethane (PU), polyethylene terephthalate (PET), polystyrene (PS), expanded polystyrene (EPS), extruded polystyrene (XPS), polycarbonate (PC), epoxy resin, acrylic, acrylonitrile butadiene styrene (ABS), polyamides (PA) (nylon), polyester (PEST), polyvinylidene chloride (PVDC) (Saran), polymethyl methacrylate (PMMA), polyacrylsulfone (PSU), polyacrylonitrile (PAN), polyvinyl alcohol (PVA), and polytetrafluoroethylene (PTFE, Teflon) [5,10].

Based on an integral and holistic approach to MP polymers, taking into account criteria such as plastic use, waste, regulations, additives, research methods, and aquatic degradation, polyester, polyamide, acrylic fibers (PES/PA/PAN), polyethylene (PE), polyethylene terephthalate (PET), polyvinyl chloride (PVC), and polystyrene (PS) were identified as the top priorities for further research into their incidence, toxicity, and health control [11]. Filter feeders, especially bivalves, are considered bioindicators of MP pollution in the marine environment. This is mainly because shellfish, especially mussels, have been successfully used as bioindicators for various monitoring purposes in the past, e.g., for the monitoring of persistent organic pollutants, heavy metals, and emerging pollutants via ‘Mussel Watch’ [12,13,14,15]. Their suitability for monitoring stems from their wide distribution in global coastal waters, sessile lifestyle, resilience, and ability to concentrate pollutants from surrounding waters, and they can occur in large populations [12,16]. The implementation of monitoring programs requires reliable standardized methods and best practice guidelines. However, optimized measurement and monitoring approaches for MPs in the environment have not yet been established. This lack has led to significant challenges in interpreting research results for use in corporate or government policy [17]. The rigorous and reproducible identification of plastic particles is crucial for pollution monitoring.

The aim of this work is to review the polymer mass fraction composition in marine bivalves and types. It highlights the need for increased attention based on factors that affect polymer abundance and their effects on environmental and human health. Recent advances in digestion steps, focusing on efficient and time-saving methods such as the microwave-assisted (MAW) procedure, are included. The application of the accurate chemical identification and quantification of MPs using pyrolysis gas chromatography–mass spectrometry (Pyr-GC-MS) is emphasized. Pyr-GC-MS is a powerful tool for the profiling of polymer mass-related MP data in environmental and food samples. Finally, the impact of MPs on human health through the consumption of shellfish is demonstrated based on the chemical hazards of polymers.

## 2. Microplastic Polymers and Marine Bivalves

In general, polymers are the macrocomponents of plastics. They are the result of polymerization reactions that take place between individual units, so-called monomers. Polymers can consist of only one type of monomer (homopolymers) or a mixture of monomers (copolymers) [18]). The analysis of MPs is based on spectroscopy (Fourier transform infrared spectroscopy, FTIR; Raman spectroscopy) or thermoanalytical methods (Pyr-GC/MS; thermal extraction–desorption–GC/MS, TED-GC/MS), resulting in two different types of data sets, composed of particle numbers or masses, both of which determine the chemical nature of MP particles [19]. To date, small MP particles and their numbers have been associated with the risk assessment of their occurrence in the environment, including human health.

While spectroscopy techniques measure the number of plastic particles, the properties of smaller plastic particles result in distinctive mass fragmentation that is easier to detect with mass-based concentrations. Mass-based concentrations are much better suited for benchmark comparisons, such as those considering ambient conditions or regulatory limits [17]. Table 1 shows the MP polymers found in shellfish worldwide. 

**Figure 1:**
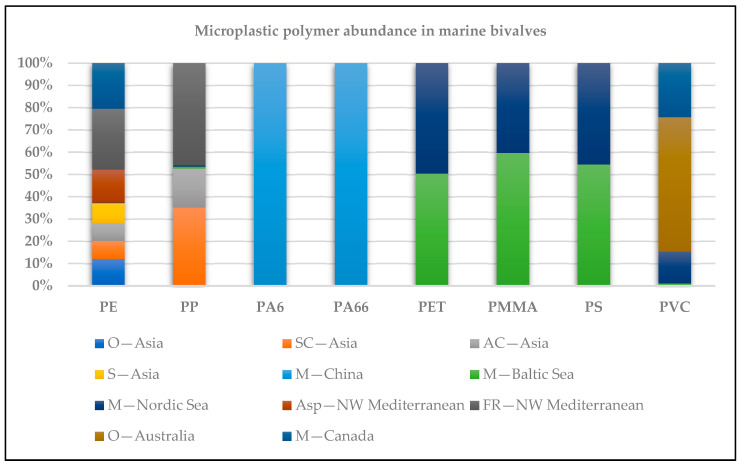
Microplastic polymer abundance in marine bivalves across the world (O, oyster; SC, Stimpson’s surf clam; AC, Asian clam; S, scallop; M, mussel; *Acanthocardia* spp.; *Fustiaria rubescens* (*Scaphopoda*).

Expressed in terms of mass quantification, Table 1 includes bivalves of economic importance, such as mussels, oysters, clams, scallops, and two benthic organisms belonging to marine bivalves (*Acanthocardia* species (spp.)) and scaphopods (*Fustiaria rubescens*) as mollusk members. The number of MP polymers quantified for most of the samples ranged from two (PA6, PA66) to nine (PA6, PC, PE, PET, PMMA, PP, PS, PVC, PUR). These are polymers of ecological significance. The polymer mass profile, expressed in µg/g wet or dry weight of the mussels studied, was characterized by the predominance of PE, PP, PVC, PET, PS, PMMA, PA66, PC, and PA6, in descending order [20,21,22,23,24,25]. MP polymers found in marine bivalves in Asia to North America are shown in Figure 1, illustrating the main polymer abundance trends. Various digestion procedures were used for sample preparation, as illustrated in Table 2.

The environmental samples of mussels and Asian oysters, clams, and scallops showed the highest mass loads [22]. The most comprehensive data are contained in the study by Halbach et al. for nine environmentally relevant polymers, in which the authors applied a retrospective approach to analyze polymer-specific MP trends in mussels from the North and Baltic Seas over three decades (since 1986 and 1992) [23]. As can be seen from Table 1, the mass of abundant polymers varies greatly in descending order for PVC, PET, PMMA, PS, and PC. Halbach et al. conclude that the different temporal trends of the polymers analyzed depend on the supraregional (PVC, PET) and regional levels (PS). PMMA occurs in constant and similar concentrations, and PC is the polymer with the lowest concentrations but with an increasing trend in recent decades [23]. The existing literature on marine exposure to MPs is almost exclusively limited to results on the counting of polymer-specific MPs particles, which can lead to different polymer clusters [26,27].

The chemical composition of MPs isolated in shellfish, which was determined using spectroscopic methods, shows the highest prevalence of polyethylene polymers (PE, LDPE, HDPE), followed by PET, PP, PA, and PS. Higher concentrations were found in farmed shellfish [28,29]. PP and expanded polystyrene (EPS) have been detected in farmed mussels, and their presence has been linked to the use of these polymers to produce ropes and buoys for mussel farming [30]. PA and PEST are also widely used in shellfish aquaculture. The MPs content in shellfish is a good indicator of MPs contamination in the aquatic environment. The presence of polymers in shellfish reflects their commercial use in all areas of human consumption. In addition, the use of plastics in aquaculture, both in equipment and packaging for the various inputs, represents a source of MPs, which account for the majority of floating marine debris around the world [31].

The predominant polymer types of MPs in mussels were PET, PE, and PP, with these three polymer types accounting for more than 80% of the total MPs in *Perna viridis* from Sri Racha Bay, Thailand [32]; *Mytillus galloprovincialis* and *Scrobicularia plana* from the Portuguese coast [33]; and *Mytillus* spp. from the Portuguese coast [32,34]. This is not surprising, as PET, PE, and PP have been widely produced and used in plastic bottles, food packaging and bags, and other plastic materials in recent decades [35], and these polymers have also been documented as the most abundant MPs in aquatic environments [36,37,38].

Looking at the composition of MPs polymer masses in bivalves and the available research data, further studies are needed to confirm the trends in polymer mass in different edible marine bivalves around the world, especially in commercially important species categorized by production type (wild or farmed). A comparative assessment of MPs polymer abundances among edible bivalve groups by morphometrics and size group is needed, as well as an examination of spatial and temporal trends. Mollusks are sensitive indicators of environmental change, especially for pollutants such as MPs, heavy metals, etc., and can be used as baseline information for further assessments of the extent of MPs polymer mass pollution in the region concerned and its impact on the mollusk trade.

## 3. Measurements and Data Analysis

### 3.1. Analytical Approaches/Techniques for the Separation and Identification of Microplastic Polymers

The steps of pretreatment, detection, and quantification in MPs analysis depend on the polymer type. MPs research is challenging due to the wide variety of characteristics of these materials, such as their sizes, shapes, and polymer types. As a result, there are no standardized methods for their separation, identification, or quantification. Overall, biological material is the most difficult matrix to work with. It is often rich in lipids that need to be removed. Usually, the lipids are saponified in the presence of alkali solutions (e.g., potassium hydroxide, KOH) [39]. The most common approach is visual identification followed by spectroscopic confirmation [40,41]. The MPs are identified based on microimaging, such as Fourier transform infrared spectroscopy (FTIR), Raman spectroscopy, and scanning electron microscopy (SEM) [42]. However, thermal methods, such as Pyr-GC/MS, thermal extraction–desorption combined with gas chromatography–mass spectrometry (TED-GC/MS), and thermogravimetric analysis coupled with differential scanning calorimetry (TGA-DSC), are currently gaining more attention due to their potential for the identification and quantification of MPs. To quantify the mass fractions and achieve the chemical characterization of plastics in environmental samples, many researchers have turned to thermoanalytical spectrometric techniques, especially Pyr–GC/MS. Sample preparation for Pyr-GC/MS is similar to the preparation for spectroscopic techniques. This includes sample pretreatment, sometimes consisting of the physical separation of plastics from the matrix (e.g., density separation) and isolation from the matrix (e.g., chemical or enzymatic digestion) [43]. In many workflows, samples are filtered in a glass filter, which is cut, folded, or crushed to fit into the pyrolysis chamber [22,23,25,44]. Accordingly, there is a need to standardize this methodology to obtain comparable results when policy-relevant work is required.

Within marine fisheries, bivalves deserve special focus due to their value but also their exposure to MPs polymers, as discussed in the next section. Digestion is the most commonly used method for MPs isolation from bivalve tissues, usually performed with a range of acids [45], enzymes [23], alkalis [46], and oxidizing agents (e.g., H_2_O_2_), with additional steps of density separation [46]. KOH is effective in breaking down biota tissue, but saponification frequently complicates traditional KOH digestion methods. In lipid-rich tissues, clogging may occur, requiring additional steps in the procedure, such as adding ethanol. Ethanol does not interfere with the MPs; however, it prolongs the reaction time to 24 h [47,48]. MPs isolation protocols require time and appropriate digestion conditions to ensure polymer integrity. The microwave-assisted (MAW) procedure has recently been identified as an economical and efficient method due to its reduced processing time and acceptable recoveries in determining MPs polymer content in marine biota. The available MAW procedures applying MsP extraction in marine biota and food are presented in Table 2.

**Table 2:** Microwave-assisted approaches for the separation of microplastic polymers in marine biota and food.

Targeted Microplastic Polymers	Matrix	Microwave-Assisted Procedure (MAD/MAE), System, and Total Microplastic Polymer Recovery (%)	Filtration Filters/Filtration Performance	Identification/ Quantification Method	Reference
PET, PS, EPS, PP, HDPE, LDPE, PC, PVC, PU	Seafood tissue (acoupa weakfish, tuna fish, trahira, shrimp)	MAD with HNO_3_; System: Ultrawave™, Milestone, Sorisole, Italy; Recovery range: 103–106%	Vacuum system filtration (2 μm linter fiber filter paper (Whatman No. 589/3) Florham Park, MI)	Gravimetry	[49]
PE, PP, PS, PMMA, PVC, PC	Mussels, shrimp, salmon fillets	MAE of GF filters in DCM after sample digestion in KOH; System: Mars6 system (CEM, Matthews, NC, USA); Recovery range: 51–151%	Matrix-dependent filters, sequential filtration with 1 μm GF filters, 20 μm and 10 μm polycarbonate filters	Pyr-GC-MS	[21]
HDPE, PP, PET, PA, PS	Dried *Mytilus galloprovincialis*	MAD multireagent digestion: KOH, H_2_O_2_, and methanol; System: Ethos system (Milestone, Sorisole, Italy); Recovery range: 99.4–100.5%	1.2 μm GF filters	Optical microscopy and Raman spectroscopy	[49]
PVC, HDPE, LDPE, PP, PS, PET	Unique food matrix (fiber, apple, courgette, cucumber, kidney beans, lettuce, potatoes, tomatoes, bread, cheese, yoghurt)	MAD with HNO_3_; System: Mars Xpress Microwave (CEM Corporation, Matthews, NC, USA); Recovery: 102.2%	3 μm Duran glass filter (VWR)	ATR FTIR	[50]
PE, PP, PS ABS, SBR, PMMA, PC, PVC, PET, N6, and N66	Mussels (commercial lyophilized flour of *Perna canaliculus*)	MAD with HCl/or HNO_3_; System: Ethos X Advanced Microwave Extraction System (Milestone Srl, Italy); Recovery: 87–138%	0.7 μm quartz fiber filter	Pyr-GC-MS	[44]

Dichloromethane (DCM); glass fiber filter (GF): hydrochloric acid (HCl); microplastic polymers: expanded polystyrene (EPS), high-density polyethylene (HDPE), low-density polyethylene (LDPE), polycarbonate (PC), polyethylene terephthalate (PET), polypropylene (PP), polystyrene (PS), polyvinyl chloride (PVC), polyurethane (PU); microwave-assisted digestion (MAD); microwave-assisted extraction (MAE); potassium hydroxide (KOH).

Several MAW approaches have been optimized to obtain quick, economic, and efficient methods for the determination of the MPs content in seafood so as to evaluate the environmental impacts generated by human actions. These optimized MAW procedures usually include the digestion of marine samples with different concentrations of HNO_3_ [44,49,50] or HCl [44] or one use of KOH together with H_2_O_2_ and methanol. Another MAW extraction procedure extracts the glass fiber filters in dichloromethane after performing KOH digestion of the initial environmental marine samples [21]. Biale et al. included the largest number of MPs polymers and, for the first time, used an MPs calibration standard mixture diluted in silicon dioxide (SiO_2_) [44]. This enabled them to evaluate the recovery of MPs after different digestion approaches, using plastic particles with sizes in agreement with those in the environment. Additional extraction improvements in the MAW digestion method are required to obtain higher extraction recoveries for PET, PA6, and PA66. The use of the MAW enables the economic and efficient isolation of the polymer content of MPs in marine biota, while avoiding the conventional, time-consuming pretreatment procedures for MPs analysis.

Instrumental analytical techniques based on analytical pyrolysis provide qualitative and mass-based quantitative information and have high potential to become of general use in the analysis of MPs. An overview of the Pyr-GC/MS method’s performance characteristics, obtained across the available research on microplastic polymers in bivalves, is given in Table 3. The specific pyrolysis products used as markers, identified through qualitative thermochemical analysis, are presented for sets of two to eleven polymers, all of environmental significance.

A Pyr-GC/MS result is obtained when an indicator ion of the marker compound is extracted, and the peak area of this ion is used for calibration. This approach is similar to the construction of calibration curves for the GC/MS quantification of organic compounds, including environmental pollutants. However, unlike in the case of a single compound analyte, it is best to confirm the presence of a polymer in a sample by identifying multiple marker compounds of this polymer. For example, the presence of PE should be validated by confirming the presence of at least five of these homologous triplet series in the C16–C26 range, even if only one or two compounds can be selected for quantification. The pyrolytic markers used for the identification and quantification of the same MPs polymer differ in the studies presented. It should be emphasized that the most significant markers useful for the identification of specific polymers are generally byproducts in polymer pyrolysates. Moreover, the yield is strongly influenced by the sample/matrix composition, as the effects of the matrix on the pyrolysis mechanisms are not negligible [51]. To improve GC performance and increase the range of MPs detectable in a single pyrolysis run, the advantages of thermally assisted hydrolysis and methylation (THM) with tetramethylammonium hydroxide (TMAH) for condensation polymers and addition polymers with oxygen-containing side chains were utilized in a study [23] on PA6, PET, PC, and PUR. Several authors have pointed out the poor recovery rates of PET [20,44], which can possibly be resolved using internal calibration, thermochemolysis (TMAH application), or the screening of possible alternative pyrolytic markers.

The studies listed in the table include polymer mass quantification, performed using external and internal calibration curves, dissolving calibration standards in a solvent, weighing individual polymer particles in an inert solid matrix, or weighing an inert solid matrix of 11 mixed polymer standards. The limit of detection (LOD) and the limit of quantification (LOQ) depend on the calibration standard preparation, the quantification marker [52], and the polymer type [53]. While individual calibration curves extend the calibration range, a drawback is that changes in relative signal intensity resulting from polymer interactions are not captured. Matsueda et al. [54] investigated this with their solvent and an inert solid matrix of 11 mixed polymer standards. Internal pyrolysis process standards (ISTDpy) have been proposed to improve quantification by avoiding fluctuations within the calibration curves due to variable organic loading [51,55]. The proposed internal standards mimic the potential interactions of the polymer-specific indicator compounds with the pyrolysis products of the remaining organic sample matrices during the pyrolysis process. The mussel samples in the reviewed Pyr-GC/MS publications were all subjected to single-shot pyrolysis analysis, where the samples were flash-pyrolyzed at a high temperature of ≥500 °C, resulting in pyrolysates that were read with a pyrogram.

Recently, “double-shot” pyrolysis analysis has been used more frequently, as it offers the possibility to separate natural organic materials from the polymer pyrolysates and improves quantification in complex matrices such as human blood [56,57] and solvent-extracted sewage sludge [58]. The “double-shot” mode involves two steps for the sequential analysis of different compounds, including volatile compounds present in the sample (low-molecular-weight substances released at lower temperatures during thermal desorption) and decomposition fragments of macromolecules that are not themselves volatile and are generated at higher temperatures during the pyrolysis step. The potential of double-shot Pyr-GC/MS lies in the characterization of volatile additives and polymers in the same sample by exploiting their thermal separation. A disadvantage of double-shot Py–GC/MS is that it is more time- and resource-intensive, taking twice as long to analyze a sample as in single-shot analysis [43]. Analytical pyrolysis characterizes MPs at the molecular level and identifies not only synthetic polymers but also the possible presence of additives. The main advantage of Py-GC-MS over the commonly applied FTIR spectroscopy is that both polymer types and organic plastic additives can be analyzed in a single analytical run, and the monitoring of additives in MPs has received increased attention due to their potential toxicity.

### 3.2. Risk Assessment Studies for Microplastic Intake in Humans Through Bivalve Consumption

The hazards posed by MPs include their physical properties (e.g., size, shape, and surface), the additives intentionally used in plastic production, their durability in the environment, and their ability to absorb chemical contaminants and pathogens and concentrate them in the food chain [5]. Much of the concern raised by MPs is due to the effects that could occur in the desorption of pollutants absorbed by the MPs themselves and the chemical additives that they contain [59,60]. The assessment of potential risks from MPs in bivalves focuses primarily on the polymer type, its frequency, and its consumption.

The risk exposure from the human consumption of bivalves (the human risk of MPs ingestion, HRI) is determined based on the abundance of MPs in shellfish (derived from the number of MPs per gram—pieces/g) and shellfish consumption data (expressed as the number of MPs ingested per day—MPs/day—or year—MPs/year) [61,62]. Another aspect to be considered is the risk posed by the plastic polymers contained in mussels—the polymer hazard index (PHI). Different polymers may pose different threats and potential health hazards based on the monomers that constitute them, which have been reviewed in the European Chemicals Agency database [63]. The PHI is calculated as

PHI = ƩPn * Sn

where PHI is the polymer risk index caused by MPs, Pn is the percentage of MPs polymer types in the bivalve sample, and Sn is the hazard value of MP polymers derived from Lithner et al. [9]. The degree of health risk of MPs based on the PHI was evaluated using hazard scores from 1 to 10,000, which were categorized into five hazard levels (I–V) [9,64]. On a monomer basis, 55 plastic polymers were graded and analyzed. The most hazardous polymers are those produced from mutagenic or carcinogenic monomers. Polymers that are ranked in this category belong to the families of PU, PVC, epoxy resins, and styrene copolymers due to their high hazard scores [9]. The accurate identification of plastic polymers in MPs samples is essential, as it underpins the assessment of the potential risks posed by these contaminants to humans and ecosystems. As shown in Table 4, the human risk from MPs intake is indicated by the presence of polymers with high hazard scores, such as PVC, PA, and PS, with hazard scores of 10,551, 47, and 30, respectively. 

This results in a hazard level of IV and a high risk category for wild mussels from the Atlantic coast of Northern Portugal; farmed and wild clams, mussels, and green mussels from Fujian Province, China; and bivalves from 14 countries, including India and Portugal. The same applies for the consumption of *Perna viridis* and *Crassostrea* spp. in India and mussels in Portugal [62,68,69]. Across the available studies presented in Table 4, countries and bivalves with a moderate risk (hazard level III) include mussels, oysters, cockles, and clams (Thailand); *Donax trunculus* (Italy, Mediterranean); and farmed and market-purchased *Sinovacula constricta* (Zhangzhou City, China). Accordingly, bivalves from six countries (Italy, China, South Africa, New Zealand, South Korea, and the USA) were identified as having moderate pollution for mussels, oysters, and clams. Furthermore, Dang et al. found that, in natural rock oysters (*Saccostrea* spp.) and sea clams (*Venus* spp.) from the South Central Coast, Vietnam, the high risk level is due to the presence of PA. PA is a dense polymer, prone to sinking and likely related to the bivalve habitat at the bottom of the sea. In the investigated bivalves from 14 countries, including those with high and moderate risks, the occurrence of PVC is confirmed. PVC, categorized as a “very high hazard” polymer, may release carcinogenic monomers and intrinsic plastic additives when entering the marine environment [67]. Some pollutants, such as persistent organic pollutants (POPs), are easily absorbed onto the surface of PVC, resulting in compound ecological effects [70]. Additionally, PVC, as a negatively buoyant plastic (density: 1.16–1.58 g/cm^3^), commonly accumulates in the deeper layers of the water or the benthic zone. Thus, infaunal bivalves, such as clams (sediments) and scallops (below 15 m), have increased potential for exposure to PVC [30]. Therefore, PVC MPs ingested by bivalves might pose a significant threat to the marine ecosystem and human health.

One of the most common pathways by which MPs enter the human body is ingestion. The first evidence of MP detection in human feces suggests that humans involuntarily ingest them, with PP (63%) and PET (17%) predominantly identified in human feces [71], coinciding with the dominant polymers detected in bivalves presented in Table 4.

Bivalves are an essential component of the human diet; therefore, contaminated bivalves containing MPs are non-negligible sources of human exposure. The HRI estimated for consumers across the world is variable, with the highest exposure level in China (8369 microplastics/capita/year) [67]. Therefore, compared to China, the risk of MP intake for other areas through the consumption of bivalves can be considered low. The distinction between farmed and wild bivalves, where available, is important to note, as farming methods can lead to varying degrees of MPs contamination.

## 4. Conclusions and Perspectives

The reviewed studies indicate the increasing microplastic pollution of marine bivalves of different habitats and geospatial origins, posing a direct risk to human health. The profile of polymer MPs isolated from marine bivalves varies. Nevertheless, the dominance of PE, followed by PVC, PET, PP, PA, PS, and PC, was observed. These observations suggest a need for the chemical identification of MPs particles in marine bivalves and other organisms to avoid the over- and underestimation of MPs, which can occur when relying solely on visual identification. Accurate identification of plastic polymers in microplastic samples may be essential, as it underpins the assessment of the potential risks posed by MPs to humans and ecosystems. Among the available methods for the isolation, identification, and quantification of microplastic polymers, approaches that offer reduced analysis times while maintaining satisfactory polymer integrity, such as microwave-assisted isolation, and the precise mass fragmentation advantages of Pyr-GC-MS, are highlighted. Based on the “One Health” principle, there is an urgent need to address the global environmental problem of MPs through the following:

- Specifically optimized, robust, and relatively rapid methods of quantifying small MPs in selected environmental samples relevant to the marine food web;
- The accurate identification of plastic polymers in microplastic samples and assessment of the potential risks posed by MPs to humans and ecosystems;
- Comprehensive monitoring and risk assessment strategies to mitigate the impacts of MPs on both food safety and marine ecosystems, as similar polymer contamination levels have been reported in marine bivalves.

## Figures and Tables

**Table 1:** Pyr-GC/MS results for microplastic contamination of bivalve samples from different parts of the world.

Bivalve Species and Sample Origin (Single Marine Shellfish Polymer Content in µg/g Wet Weight)							
	*Crassostrea gigas* Australian Local Fish Market	Mussel (*Mytillus* spp.) Environmental Samples	*Acanthocardia* spp. Northwestern Mediterranean	*Fustiaria rubescens* (*Scaphopoda*) Northwestern Mediterranean	*M. edulis* Baltic Sea (BS), North Sea (NS)	Commercially Available Mussels Chinese Seafood Market	Oysters, Stimpson’s Surf Clams, Asian Clams, Scallops
Investigated polymers	PS, PMMA, PVC, PE, PET, PP	PC, PE, PMMA, PP, PS, PVC	PC, PE, PET, PMMA, PP, PS	PC, PE, PET, PMMA, PP, PS	PA6, PC, PE, PET, PMMA, PP, PS, PVC, PUR	PA6, PA66	PE, PET, PMMA, PP, PS, PVC
PA66	- **	-	-	-	-	0.2	-
PA6	-	-	-	-	-	0.06	-
PC	-	<5.8	-	-	0.002 *–0.495 * (BS) 0.001 *–0.43 * (NS)	-	-
PE	not detected	18.0–32.9	92 *	169 *	0.45 *–5.83 * (BS) 0.03 *–2.69 * (NS)	-	10–15
PET	-	-	-	-	0.13 *–5.09 * (BS) 0.05 *–10.39 * (NS)	-	not detected
PVC	<10.93–23.5	<2.3–41.6	-	-	0.33 *–9.84 * (BS) 0.59 *–7.61 * (NS)	-	not detected
PMMA	not detected	not detected	-	-	0.06 *–2.99 * (BS) 0.09 *–1.12 * (NS)	-	not detected
PS	not detected	not detected	-	-	0.03 *–2.44 * (BS) 0.09 *–1.12 * (NS)	-	not detected
PP	<2.45	<1.2		13 *	0.04 *–1.54 * (BS) 0.02 *–0.87 * (NS)	-	not detected
Reference	[20]	[21]	[22]	[22]	[23]	[24]	[25]

* µg/g dry weight; **—not investigated; µg/g ww (wet weight); polyamide (PA), polycarbonate (PC), polyethylene (PE), polyethylene terephthalate (PET), polyvinyl chloride (PVC), polymethylmethacrylate (PMMA), polystyrene (PS), polypropylene (PP), polyurethane (PU); pyrolysis–gas chromatography–mass spectrometry (Pyr-GC/MS).

**Table 3:** Overview of Pyr-GC/MS method’s performance characteristics for identification and quantification of MP polymer composition in bivalves.

Bivalve Species	Characteristic Compound (Decomposition Product)/Quantitative Ions (m/z)	Calibration Curve	Internal Standard	Linear Range (µg)	Recovery Range (%)	LOQ (µg)	Reference
Oyster (*Crassostrea gigas)*	PE: 1-Decene (C10), 3-tetradecne (NIST) or 1-dodecene (C12), 1-tetradecane (C14), m/z = 83 PET PMMA: Methylmethacrylate, m/z = 100 PP: 2,4,6-Dimethyl-1-heptene, m/z = 126 PS: Styrene dimer: 3-butene-1,3-diyldibenzene, m/z = 130 PVC: Benzene, m/z= 78	External, ASE extraction of solid standards	-	0.02–10	Expressed as RSD: 3–12	0.96–24.29	[33]
Mussel (*Mytilus* spp.)	PC: 4-Isopropenylphenol, m/z = 134 PE: Alkene C17 peak, m/z = 83 PMMA: Methyl-methacrylate, m/z = 100 PP: 2,4-Dimethyl-1-heptene, m/z = 70 PS: 3-Butene-1,3-diyldibenzene (styrene trimer), m/z = 91 PVC: Indene, m/z = 115	Internal, MAE dissolution of solid standards	Styrene-d5 (m/z = 109)	0.5–10 (PE, PVC) 0.25–5 (PP, PS, PMMA, PC)	Mean recovery: 51.1–150.9%	1.2 (PP, PS)–5.8 (PC)	[34]
*Acanthocardia* spp. *Fustiaria rubescens*	PC: Methyl-bisphenol A, 241- > 133 (quantification), 256-> 241 (confirmation) PE: 1,12-Tridecadiene, 95- > 67 (quantification), 109- > 67 (confirmation) PET: Dimethylterephthalate, 163- > 135 (quantification), 163- > 103 (confirmation) PMMA: Methylmethacrylate, 100- > 41 (quantification), 100- > 69 (confirmation) PP: Dimethyleheptane, 70- > 55 (quantification), 126- > 83 (confirmation) PS: Styrene trimer, 207- > 129 (quantification), 207- > 71 (confirmation)	External, solid cryo-milled polymers diluted in a calcined powdered glass microfiber filter		0.025–1.360	Recovery: 82 –129%	0.13 ^AL^	[35]
*M. edulis*	PA6: ε-Caprolactam, m/z = 113 N-methyl caprolactam *, m/z = 127 PC: 2,2-Bis(4’-methoxyphenyl)propane *, m/z = 241 PE: α,ω-Alkanes (e.g., C20), m/z = 82 PET: Dimethylterephthalate*, m/z = 163 PMMA: Methylmethacrylate, m/z = 100 PP: 2,4-Dimethylhept-1-ene, m/z = 70 PS: 2,4,6-Triphenyl-1-hexene, m/z = 91 PVC: Naphthalene, m/z = 128 PUR: 4,4’-Methylenbis(N,N-dimethylaniline) *, m/z = 254	Internal, solid standards	4 internal standards: 9-tetradecyl-1,2,3,4,5,6,7,8-octahydro anthracene (TOHA), deuterated anthracene, cholanic acid, and deuterated polystyrene	0.5 to 50 μg, 4 internal standards (9-tetradecyl-1,2,3,4,5,6,7,8-octahydro, 0.5 µg; cholanic acid, 0.5 µg; anthracene (d10), 1 µg; polystyrene (d8), 1 µg)	-	0.3–1.4	[20]
Commercially available mussels	PA66: Cyclopentanone, m/z = 84 PA6: Caprolactam, m/z = 113	External, dissolved standards	-	2–64	Average recovery: 81.5–94.5	0.6 (PA6)–2.0 (PA66)	[36]
Oysters, Stimpson’s surf clams, Asian clams, scallops	PE: 1-Undecene, m/z = 55 PET: Biphenyl, m/z = 154 PMMA: Methylmethacrylate, m/z = 100 PP: 2,4-Dimethylhept-1-ene, m/z = 70 PVC: Naphthalene, m/z = 128 PS: Styrene, m/z = 104	External, solid standards	-	4.4–16. 27	Recovery: 82–85	-	[37]
Mussels (commercial lyophilized flour of *Perna canaliculus*)	ABS: 2-Phenethyl-4- phenylpent-4-enenitrile (SAS), m/z = 170 PA6: Caprolactam, m/z = 133 PA66: Cyclopentanone, m/z = 84 PC: Bisphenol A (BPA), m/z = 213 PE: α,ω-Alkanes C15–C25 (average of the areas), m/z = 82 PET: Benzoic acid (BA), m/z= 122 PMMA: Methylmethacrylate (MMA), m/z = 100 PP: 2,4-Dimethyl-1-heptene, m/z = 126 PS: 3-Butene-1,3-diyldibenzene (styrene dimer), m/z = 91 SBR: Butadiene trimer, m/z = 79	Internal, solid standard diluted in SiO2	Anthracene-d10	0.3–39	Recovery: 87–138	0.003–0.04	[43]

Accelerated solvent extraction (ASE); analytical limit of detection (AL); dichloromethane (DCM); microwave-assisted extraction (MAE); [m/z] = mass to charge ratio; n-C16-C26-alkadienes used for quantification of PE; * only after TMAH treatment; acrylonitrile butadiene styrene (ABS), polyamide (PA6, PA 66)), polycarbonate (PC), polyethylene (PE), polyethylene terephthalate (PET), polyvinyl chloride (PVC), polymethylmethacrylate (PMMA), polystyrene (PS), polypropylene (PP), polyurethane (PU), styrene-butadiene rubber (SBR); pyrolysis–gas chromatography–mass spectrometry (Pyr-GC/MS).

**Table 4:** Summarized estimates of the risk of human ingestion of microplastics and the polymer hazard index and hazard level (risk category) due to MPs in bivalves.

Shellfish Species/Location	Human Risk of Microplastic Intake, HRI (MPs/Day)/(MPs/Year)	Dominant Polymers	Polymer Hazard Index Caused by MPs (PHI)	Hazard Level/Risk Category	Reference
Wild mussels (*Mytilus Galloprovincialis*), Portugal	2-6/680–2342	PE, PA, PS, PP, PEVA	1505–2850	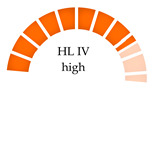	[62]
Farmed bivalves: Green mussels (*Perna viridis*), oysters (*Saccostrea cucullata*), cockles (*Tegillarca granosa*), and clams (*Meretrix meretrix*), coastal areas of Thailand	0.52 (MPs/day)	PET, cotton as cellulosic polymer, rayon, PP, PE, PP, acrylic, PA, PS	149–964	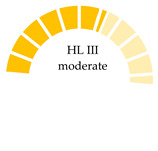	[65]
*Donax trunculus*, Italy—Tuscany Coast (Mediterranean Sea)	19.2 (MPs/year)	PE, PET	NR	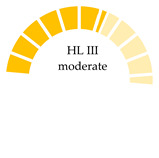	[66]
Farmed and market samples of *Sinonovacula constricta*, Zhangzhou City, China	NA	PP, PE, PA, PET, PC	623 (farmed)–819 (market samples)	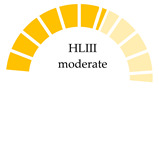	[67]
Mussels (*Mytilus galloprovincialis*), Portugal	7/2438–2650	Cellulose, polyacrylonitrile, PP, PS, PA, PET, LDPE	NA	NA	[61]
Bivalves from 14 countries across the world	0–8369 (MPs/year)	PVC, PET, PE, PS, PMMA, PA, PP	220– 12,050	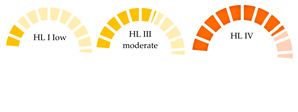	[68]
Farmed and wild samples: Clams (*Meretrix meretrix*), mussels (*Mytilus edulis*), green mussels (*Perna viridis*), Fujian Province, China	NA	PET, PAN	3813–3928 (farmed) 3925–4478 (wild)	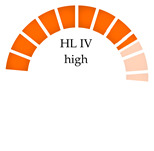	[69]

Not analyzed (NA); not reported (NR); microplastic polymers: polyamide (PA), polyacrylonitrile (PAN), polyethylene vinyl acetate (PEVA), polycarbonate (PC), polyethylene (PE), low-density polyethylene (LDPE), polyethylene terephthalate (PET), polyvinyl chloride (PVC), polymethylmethacrylate (PMMA), polystyrene (PS), polypropylene (PP), polyurethane (PU); polymer hazard index (PHI). Polymer risk is categorized as follows: hazard level I (PHI < 10) = low risk; hazard level II (PHI: 10–100) = medium risk; hazard level III (PHI: 100–1000) = moderate risk; hazard level IV (PHI:1000–10 000) = high risk; and hazard level V (PHI > 10 000) = very high risk.

## Data Availability

No new data were created or analyzed in this study.

## Abbreviations

The following abbreviations are used in this manuscript:

Pyr-GC/MS	pyrolysis gas chromatography–mass spectrometry
TED-GC/MS	thermal extraction–desorption combined with gas chromatography–mass spectrometry
TGA-DSC	thermogravimetric analysis coupled with differential scanning calorimetry
PP	polypropylene
PE	polyethylene
PVC	polyvinyl chloride
PS	polystyrene
HDPE	high-density polyethylene
LDPE	low-density polyethylene
PET	polyethylene terephthalate
EPS	expanded polystyrene
XPS	extruded polystyrene
PC	polycarbonate
ABS	acrylonitrile butadiene styrene
PA	polyamides
PEST	polyester
PVDC	polyvinylidene chloride
PMMA	polymethyl methacrylate
PSU	polyarylsulfone
PAN	polyacrylonitrile
PVA	polyvinyl alcohol
PTFE	polytetrafluoroethylene
